# Editorial for Special Issue “Nano-Bioremediation Approaches for Degraded Soils and Sustainable Crop Production”

**DOI:** 10.3390/nano13071138

**Published:** 2023-03-23

**Authors:** Vishnu D. Rajput, Tatiana Minkina

**Affiliations:** Academy of Biology and Biotechnology, Southern Federal University, Rostov-on-Don 344090, Russia; tminkina@mail.ru

In recent decades, the global population has rapidly increased, resulting in an increasing demand for food. However, due to enhanced technogenic and industrial activities, the cultivable land area is either decreasing or becoming polluted with toxic elements that hamper sustainable crop production and impose a threat to food security. To achieve the goal of zero hunger, it is necessary to change conventional approaches to remediating or restoring degraded soil. Nanomaterials are a fast-expanding group of materials with a wide array of properties and have shown promising results in soil health and crop improvement. However, their application at polluted sites still needs to be intensively explored. This Special Issue on nano-bioremediation approaches for degraded soils and sustainable crop production aims to combine related research to explore the realistic implementation of nano-enhanced remediation and sustainable crop production. A total of seven scientific articles, including five reviews and two research papers, have been published in this issue on the mitigation of abiotic stresses and impacts of climate change to ensure sustainable food security [1], the use of biopolymeric nanopesticides to control insect-pest [2], the use of nano-fertilizers to improve nutrient use efficiency [3], bioimaging approaches to monitoring crop growth [4], the restoration of polluted soils [5], degrading dyes and other soil pollutants [6] and the remediation of wastewater using green nano-based approaches [7]. These experimental findings found that the use of immobilized microbial cells and biogenic nanomaterials, especially in the bioreactor system, could remediate contaminated industrial effluents in a cost-effective way. Furthermore, the integration of IoT, AI, 5G communication, and nanomaterials could resolve the global concern of drinking water scarcity. The nanoscale regulation process supports the adsorption and deterioration of pollutants and could absorb/adsorb a large variety of contaminants and catalyze reactions; this remediation process reduces the accumulation of pollutants while limiting their spread from one medium to another. Combining nanomaterials with phytoremediation and bioremediation methods showed promising results in the eco-friendly decontamination of polluted soils. However, most of the available research on nano-enhanced remediation is either limited to laboratory conditions or based on computational modelling. Thus, further results are required for the successful implementation of these approaches/methods. The use of nano-fertilizers or nano-enhanced products in agriculture undoubtedly improves soil health and enhances the plant growth performance. However, long-term experimental results are still required and the fate of nanomaterials in agriculture has not been well explored. Safety regulations must be followed before using nano-enhanced products in agriculture or remediation programs. Emphasis should be placed on the use of green or biogenic nanotechnology.

## References

[1] Khalid M.F., Iqbal Khan R., Jawaid M.Z., Shafqat W., Hussain S., Ahmed T., Rizwan M., Ercisli S., Pop O.L., Alina Marc R. (2022). Nanoparticles: The Plant Saviour under Abiotic Stresses. Nanomaterials.

[2] Kumar R., Kumar N., Rajput V.D., Mandzhieva S., Minkina T., Saharan B.S., Kumar D., Sadh P.K., Duhan J.S. (2022). Advances in Biopolymeric Nanopesticides: A New Eco-Friendly/Eco-Protective Perspective in Precision Agriculture. Nanomaterials.

[3] Verma K.K., Song X.-P., Joshi A., Tian D.-D., Rajput V.D., Singh M., Arora J., Minkina T., Li Y.-R. (2022). Recent Trends in Nano-Fertilizers for Sustainable Agriculture under Climate Change for Global Food Security. Nanomaterials.

[4] Pete A.M., Ingle P.U., Raut R.W., Shende S.S., Rai M., Minkina T.M., Rajput V.D., Kalinitchenko V.P., Gade A.K. (2023). Biogenic Synthesis of Fluorescent Carbon Dots (CDs) and Their Application in Bioimaging of Agricultural Crops. Nanomaterials.

[5] Rajput V.D., Minkina T., Upadhyay S.K., Kumari A., Ranjan A., Mandzhieva S., Sushkova S., Singh R.K., Verma K.K. (2022). Nanotechnology in the Restoration of Polluted Soil. Nanomaterials.

[6] Kumar P., Dixit J., Singh A.K., Rajput V.D., Verma P., Tiwari K.N., Mishra S.K., Minkina T., Mandzhieva S. (2022). Efficient Catalytic Degradation of Selected Toxic Dyes by Green Biosynthesized Silver Nanoparticles Using Aqueous Leaf Extract of *Cestrum nocturnum* L.. Nanomaterials.

[7] Malik S., Dhasmana A., Preetam S., Mishra Y.K., Chaudhary V., Bera S.P., Ranjan A., Bora J., Kaushik A., Minkina T. (2022). Exploring Microbial-Based Green Nanobiotechnology for Wastewater Remediation: A Sustainable Strategy. Nanomaterials.

